# Supplementary material for: SLIT2/ROBO1-signaling inhibits macropinocytosis by opposing cortical cytoskeletal remodeling
Source: Nat Commun. 2020 Aug 17;11:4112. doi: 10.1038/s41467-020-17651-1 (PMC7431850; doi:10.1038/s41467-020-17651-1)
Supplement: Supplementary file 1 — Supplementary Information [file 41467_2020_17651_MOESM1_ESM.pdf]

Supplementary Information

SLIT2/ROBO1 signaling inhibits macropinocytosis by opposing cortical cytoskeletal remodelling

Vikrant K. Bhosle, Tapas Mukherjee, Yi-Wei Huang, Sajedabanu Patel, Bo Wen (Frank) Pang, Guang-Ying Liu, Michael Glogauer, Jane Y. Wu, Dana J. Philpott, Sergio Grinstein, Lisa A. Robinson

Supplementary Table 1 Endotoxin levels in recombinant Slit2 preparations

Three independent preparations of recombinant Slit2 proteins (NSlit2, CSlit2 and Slit2ΔD2) were tested for endotoxin using a Toxinsensor™ Chromogenic LAL Endotoxin Assay Kit (GenScript, Piscataway, NJ, USA).

Recombinant Slit2 protein name	Purified protein concentration (µg/ml)	Endotoxin levels (EU/ml)	Endotoxin levels (EU/mg of protein)
NSlit2	50	0.0308	0.616
CSlit2	43	0.0242	0.563
Slit2ΔD2	65	0.0341	0.525
NSlit2	48	0.0253	0.527
CSlit2	70	0.0299	0.427
Slit2ΔD2	32	0.0157	0.491
NSlit2	55	0.0299	0.544
CSlit2	50	0.0234	0.468
Slit2ΔD2	35	0.0156	0.488

Supplementary Table 2 Antibody Information

Antibody Target	Source	Host/Type	Clone	Application
Robo1	Thermo Fisher Scientific	Rabbit polyclonal IgG	-	Western Blot (WB) – 1:1000 in 5% milk overnight at 4°C
Beta actin	Thermo Fisher Scientific	Mouse monoclonal IgG2b	-	WB – 1:3000 in 5% milk for 1 h at room temperature (RT)
RhoA	Cytoskeleton	Mouse monoclonal IgG1	7F1.E5	WB – 1:1000 in 5% milk for 3 h at RT
Total NF-κB p65	Cell Signaling Technology	Rabbit monoclonal IgG	C22B4	WB - 1:1000 in Superblock blocking buffer overnight at 4°C
Phospho- NF-κB p65 (Ser536)	Cell Signaling Technology	Rabbit monoclonal IgG	93H1	WB - 1:1000 in Superblock blocking buffer 1 h at RT
Total Fyn	Cell Signaling Technology	Rabbit Polyclonal IgG	-	WB - 1:1000 in Superblock blocking buffer overnight at 4°C
Phospho-Fyn (Tyr530)	Sigma-Aldrich	Rabbit polyclonal IgG	-	WB - 1:1000 in Superblock blocking buffer 1 h at RT
Total Src	Cell Signaling Technology	Rabbit polyclonal IgG	-	WB - 1:2000 in Superblock blocking buffer overnight at 4°C
Phospho-Src (Tyr416)	Cell Signaling Technology	Rabbit polyclonal IgG	-	WB - 1:2000 in Superblock blocking buffer 1 h at RT
srGAP2	Abcam	Rabbit monoclonal IgG	EP2506(2)	WB – 1:1000 in 5% milk overnight at 4°C
c-Myc tag	Abcam	Mouse monoclonal IgG1	9E10	Immunofluorescence (IF) – 1:200 overnight at 4°C
CD68	BioLegend	Rat anti-mouse IgG2a (Alexa Fluor 488 conjugated)	FA11	IF – 1:500 in 5% goat serum overnight at 4°C

Supplementary Table 3 PCR Primer Information – All primers are for murine cDNA

Gene Symbol	Primer type	Primer Sequence (5' - 3')	PCR Product size
Robo1	Forward	CAGACACCGCAAGAAGAGAA	108
Robo1	Reverse	GCTTCGCCTCCTCTTTGATAA	
Robo2	Forward	CAGTGTTTCCTGGGATCCTCCA	152
Robo2	Reverse	CAGGGAACAAGCCACCTATT	
Trio	Forward	GTACAGGAAGCCACGAGAAGG	116
Trio	Reverse	CGCACGTGACCTCACTCAAT	
Gapdh	Forward	TGGTGAAGGTCGGTGTGAAC	153
Gapdh	Reverse	CGTGAGTGGAGTCATACTGG	

Supplementary Figure- 1

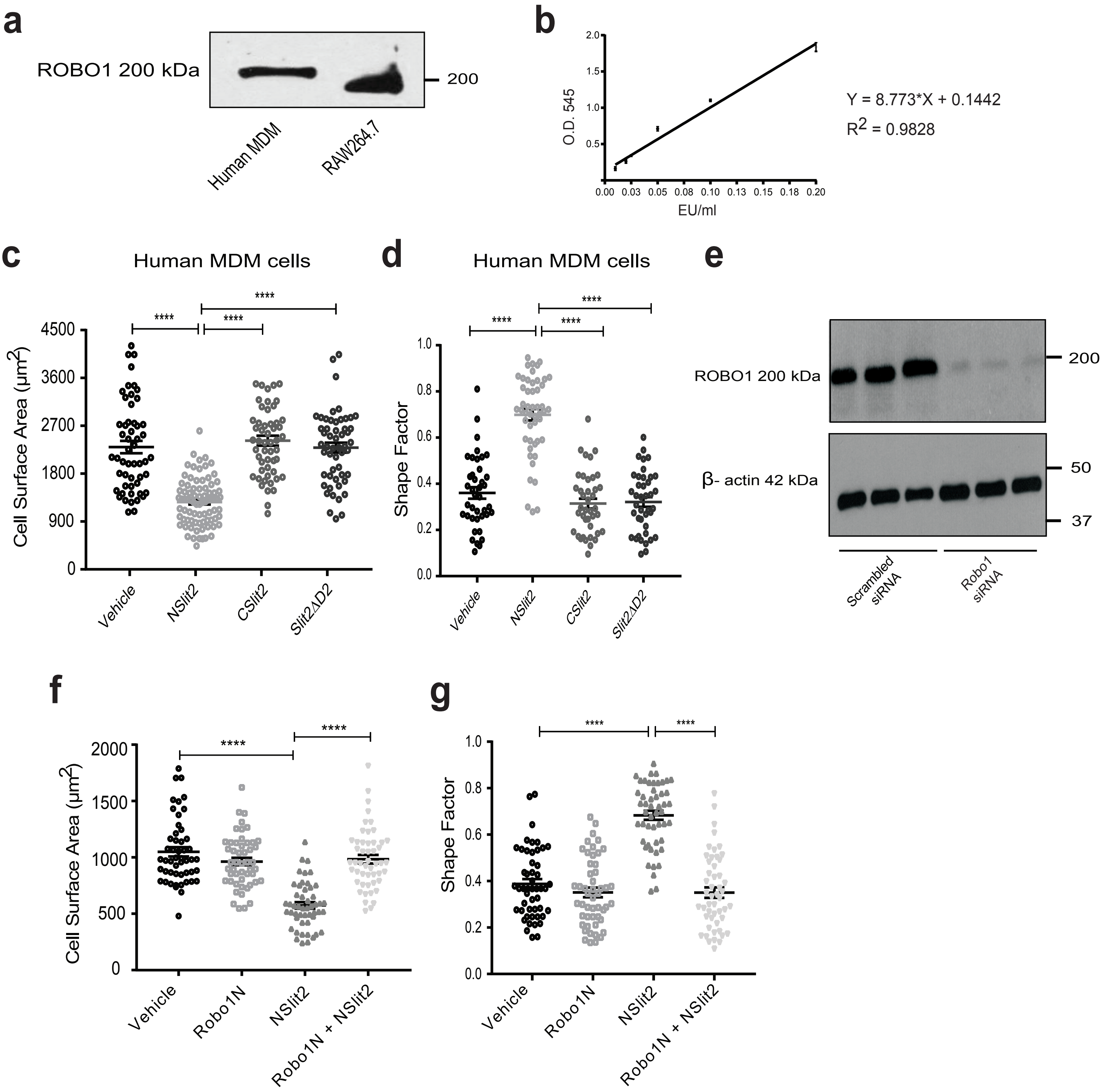

Supplementary Figure 1

(**a**) ROBO1 protein was detected in primary macrophages derived from human peripheral blood mononuclear (MDM) cells and RAW264.7 murine macrophages by immunoblotting. (**b**) Endotoxin levels were measured in recombinant NSlit2, CSlit2 and Slit2ΔD2 preparations using a chromogenic LAL Endotoxin assay kit. The standard curve was drawn using known endotoxin concentrations in the range of 0.01-1 EU/ml. Endotoxin levels in three different batches of NSlit2, CSlit2 and Slit2ΔD2 preparations are provided in Supplementary Table- 1. (**c, d, f, g**) Cell surface area and shape factor were measured using Volocity 6.3 software. Data are presented as mean ± standard error of mean (SEM). Comparisons between groups were made by one-way analysis of variance (ANOVA), followed by post hoc Tukey's multiple comparison tests. n= 50 cells per treatment group per experiment over 3 independent experiments. (**c**) Experiments were conducted as described in Fig. 1c using primary human MDM cells. **** $p <$ 0.0001, NSlit2 vs vehicle, CSlit2, or Slit2ΔD2. (**d**) Shape factor for cells in (c) was measured. **** $p < 0.0001$, NSlit2 vs vehicle, CSlit2, or Slit2ΔD2. (**e**) RAW264.7 cells were transfected with either scrambled siRNA or siRNA targeting murine *Robo1* in three independent replicates of each treatment. After 72 h, total protein lysate was collected from cells and immunoblotted for ROBO1. β-actin was used as a loading control. (**f**) NSlit2 was pre-incubated with soluble Robo1N protein in molar ratio of 1:3 for 1 h at 37°C before adding to cells, where indicated. Experiments were conducted as described in Fig. 1c using RAW264.7 cells. **** $p < 0.0001$ for the indicated comparisons and $p = 0.9761$, Robo1N vs Robo1N + NSlit2. (**g**) Shape factor for cells in (f) was measured. **** $p < 0.0001$ for the indicated comparisons and $p > 0.9999$, Robo1N vs Robo1N + NSlit2. Source data for (**a, c-e)** are provided as a Source Data file.

Supplementary Figure- 2

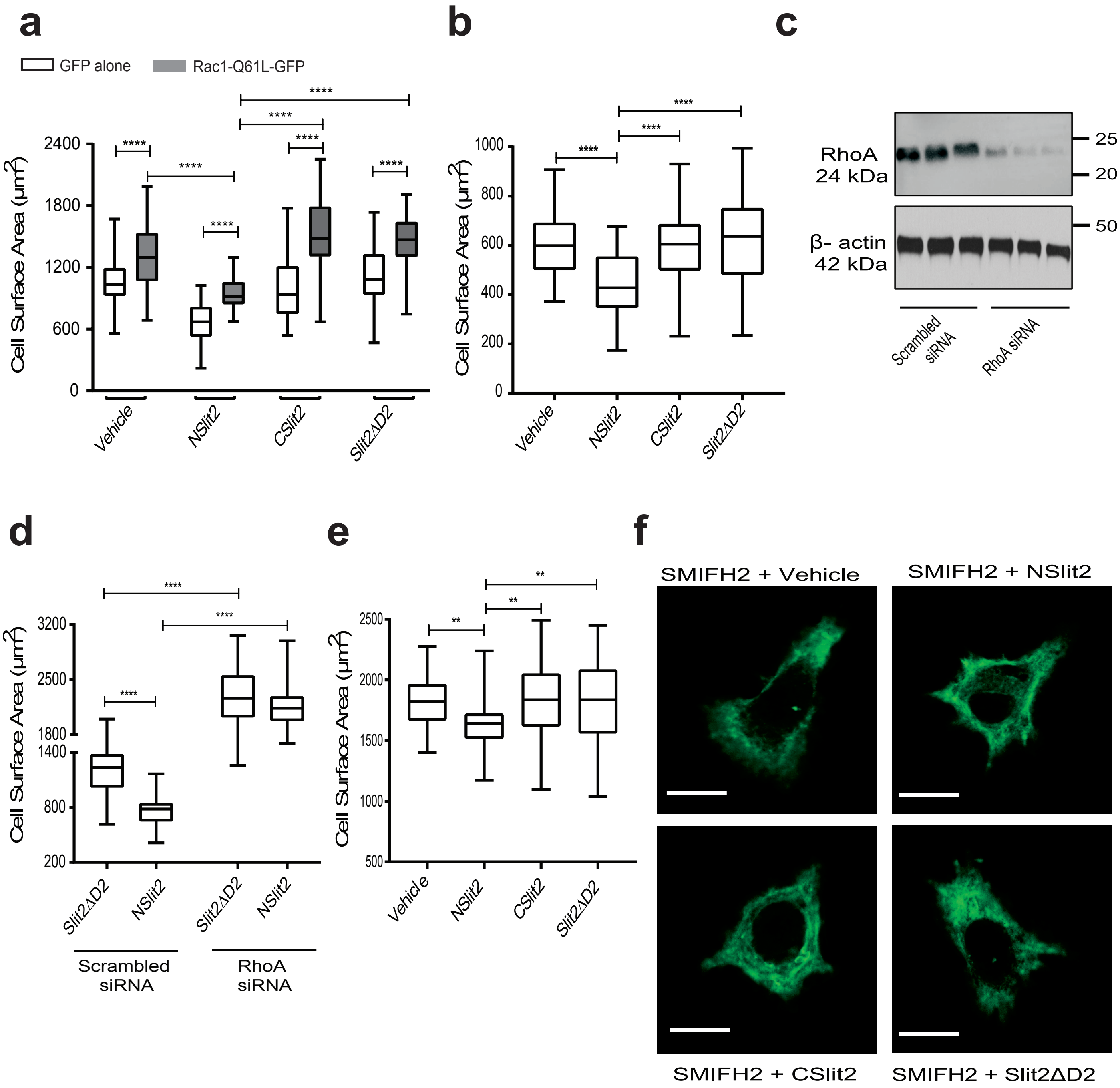

Supplementary Figure 2

(**a, b, d, e**) Cell surface area and shape factor were measured using Volocity 6.3 software. Data are presented as boxplots where the middle line is the median; lower and upper hinges correspond to the first and third quartiles; whiskers represent minimum and maximum values. Comparisons between the groups were made by two-way ANOVA in (**a**), and one-way ANOVA in (**b, d, e**) followed by post hoc Tukey's multiple comparison tests. n= 50 cells per treatment group per experiment over 3 independent experiments. (**a**) The constitutively active RAC1 (Rac1-Q61L-GFP) plasmid or control GFP plasmid were transiently expressed in RAW264.7 macrophages. After 48 h, experiments were performed as described in Fig. 1c. **** $p < 0.0001$, Rac1-Q61L-GFP vs GFP and **** $p < 0.0001$, NSlit2 vs vehicle, CSlit2, or Slit2ΔD2. (**b**) RAW264.7 cells were incubated with the Arp2/3 inhibitor, CK-666, for 60 min and cell spreading assays were performed as in Fig. 1c. **** $p < 0.0001$, NSlit2 vs vehicle, CSlit2, or Slit2ΔD2. (**c**) RhoA expression was knocked down in RAW264.7 macrophages using a specific siRNA. After 72 h, total protein lysate was collected from cells and immunoblotted for RhoA. β-actin was used as an internal loading control. (**d**) RhoA was silenced as in (c), and cell spreading assays were performed as in Fig. 1c. **** $p < 0.0001$ for the indicated comparisons and $p = 0.3350$, NSlit2 vs Slit2ΔD2 in RhoA knockdown conditions. (**e**) RAW264.7 cells were pre-incubated with the ROCK1/2 inhibitor, Y-27632 for 30 min and experiments performed as in Fig. 1c. ** $p = 0.0040$, 0.0089, and 0.0045 for NSlit2 vs vehicle, CSlit2, Slit2ΔD2 respectively. (**f**) RAW264.7 cells were treated with the formin inhibitor, SMIFH2, for 30 min and experiments conducted as in Fig. 1c. Scale bar, 25 μm. Source data for (**a-e**) are provided as a Source Data file.

Supplementary Figure- 3

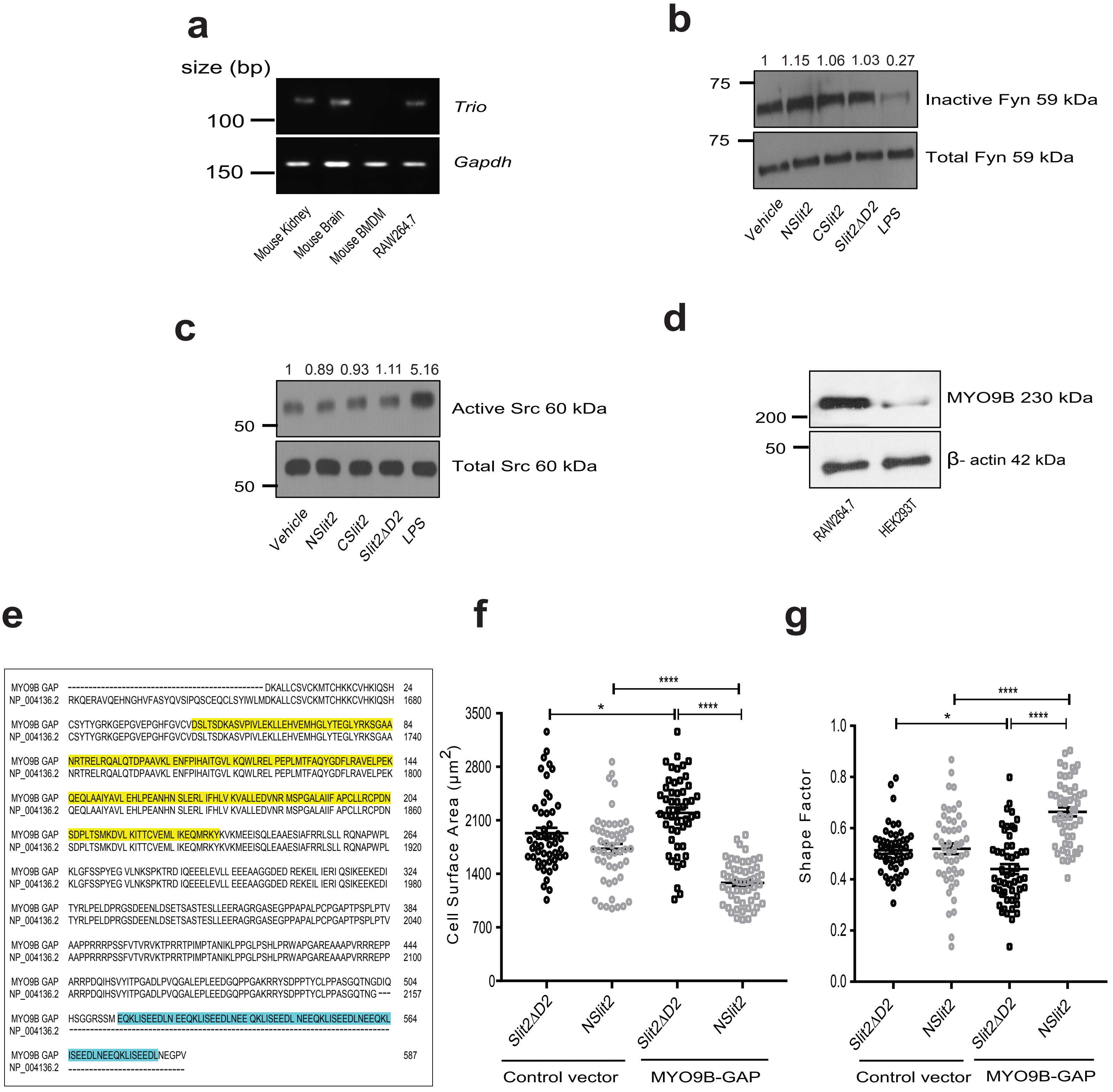

Supplementary Figure 3

(**a**) Total RNA was isolated from murine brain and kidney, as well as from RAW264.7 cells and primary murine BMDM. *Trio* mRNA expression was investigated by RT-PCR. *Gapdh* was used as a loading control. (**b**) RAW264.7 cells were treated with vehicle, NSlit2 or Slit2ΔD2 for 15 min or with LPS (0.5 mg/ml) for 30 min at 37°C. Total protein lysates were collected and immunoblotted for inactive phospho-Fyn (phospho-Tyr530) and total Fyn levels. The ratio of phospho- to total Fyn levels, relative to vehicle, is indicated for each treatment. (**c**) RAW264.7 cells were treated as described in (b). Lysates were immunoblotted for inactive phospho-Src (phospho-Tyr418) and total Src levels. The ratio of phospho- to total Src levels, relative to vehicle, is indicated for each treatment. (**d**) MYO9B protein was detected in RAW264.7 macrophages and HEK293T cells by immunoblotting. β-actin was used a loading control. (**e**) The protein product encoded by c-myc-tagged MYO9B-GAP cDNA plasmid was aligned with human myosin IXB isoform I protein (accession number - NP_004136.2). The RhoGAP domain is highlighted in yellow and the c-myc tag in blue. (**f, g**) Cell surface area and shape factor were measured using Volocity 6.3 software. Data are presented as mean ± SEM. Comparisons between the groups were made by one-way ANOVA, followed by post hoc Tukey's multiple comparison tests. n= 50 cells per treatment group per experiment over 3 independent experiments. (**f**) The c-myc-tagged MYO9B-GAP cDNA plasmid was expressed in HEK293T cells with stable expression of ROBO1 and after 48 h experiments were performed as described in Fig. 3a. * $p = 0.0137$, MYO9B-GAP Slit2ΔD2 vs control vector Slit2ΔD2 and **** $p < 0.0001$, MYO9B-GAP NSlit2 vs MYO9B-GAP Slit2ΔD2. (**g**) Experiments were performed as in (f) and cell rounding was assessed using shape factor analysis. * $p = 0.0215$, MYO9B-GAP Slit2ΔD2 vs control vector Slit2ΔD2 and **** $p < 0.0001$, MYO9B-GAP NSlit2 vs MYO9B-GAP Slit2ΔD2. Source data for (**a-d, f, g**) are provided as a Source Data file.

Supplementary Figure- 4

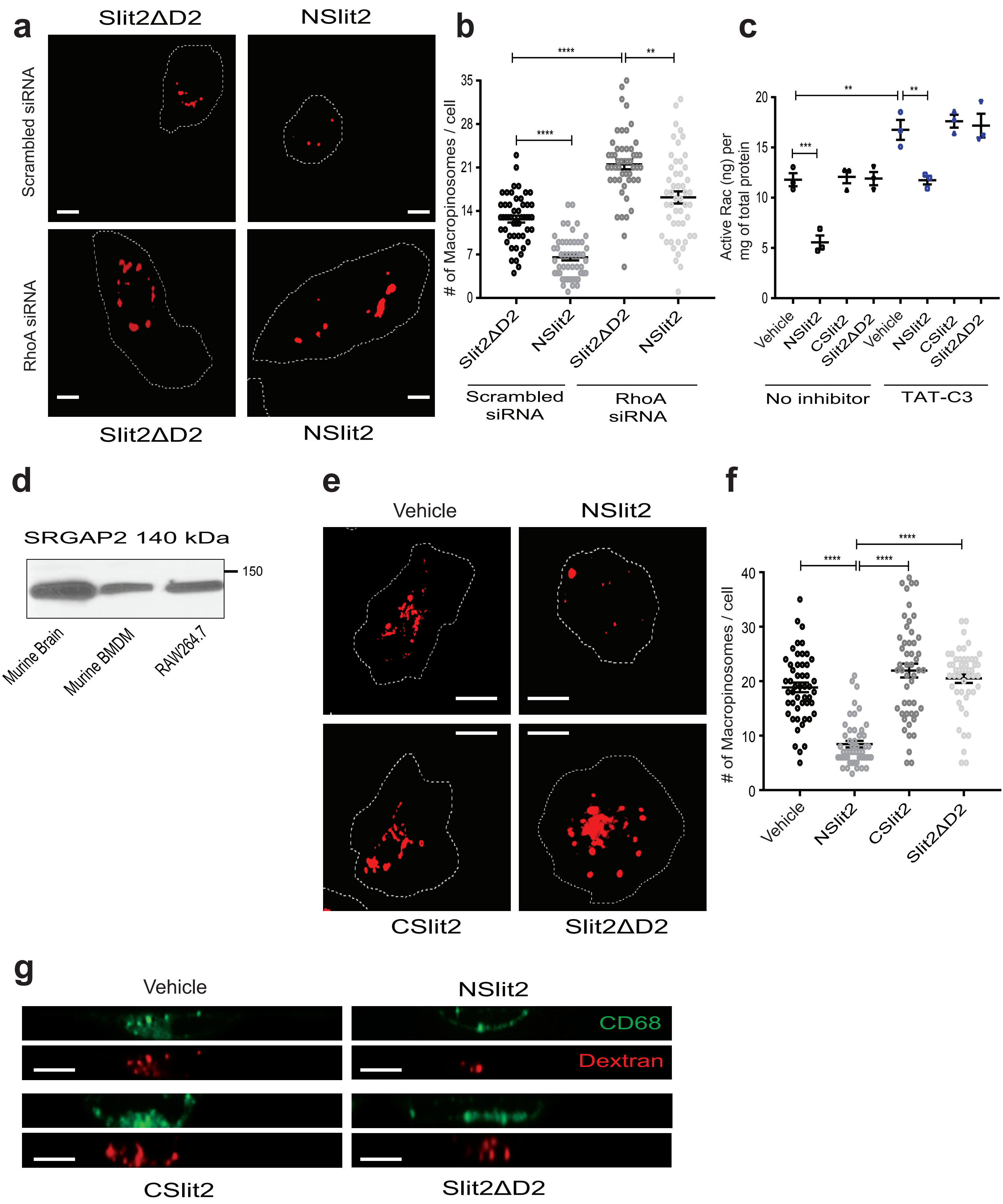

Supplementary Figure 4

(**a**) RhoA expression was knocked down in RAW264.7 cells using a specific siRNA and experiments performed as described in Fig. 4c. Scale bar, 10 µm. (**b, f**) Macropinosomes (number and size) were measured using ImageJ software, version 1.51v. All data are presented as mean ± SEM. Comparisons between groups were made by Kruskal–Wallis ANOVA, followed by Dunn's multiple comparisons test. n= 50 cells per treatment group per experiment over 3 independent experiments. (**b**) Macropinosomes were counted for cells in (a). **** $p < 0.0001$, scrambled siRNA Slit2ΔD2 vs RhoA siRNA Slit2ΔD2; **** $p < 0.0001$, scrambled siRNA NSlit2 vs scrambled siRNA Slit2ΔD2 and ** $p = 0.0030$, RhoA siRNA NSlit2 vs RhoA siRNA Slit2ΔD2. (**c**) RAW 264.7 cells were incubated with serum-free medium or the RhoA/B/C inhibitor, TAT-C3, for 4 h and then incubated with vehicle, NSlit2, CSlit2 or Slit2ΔD2 for an additional 15 min at 37°C. Active Rac1/2/3 levels were measured using a calorimetric G-LISA assay kit. *** $p = 0.0006$, No inhibitor, NSlit2 vs vehicle; ** $p = 0.0059$, No inhibitor vs TAT-C3; and ** $p = 0.0054$, TAT-C3 NSlit2 vs TAT-C3 vehicle. (**d**) Total protein lysate from murine brain, primary murine BMDM and RAW264.7 cells was isolated and immunoblotting performed to detect SRGAP2 protein. (**e**) RAW264.7 cells were pre-incubated with vehicle, NSlit2, CSlit2, or Slit2ΔD2 for 10 min followed by co-incubation with murine CSF1 for an additional 15 min. Cells were incubated with TMR-labeled 70kDa dextran as in Fig. 4a. Scale bar, 10 µm. (**f**) Macropinosomes were counted for cells in (e). **** $p < 0.0001$, NSlit2 vs vehicle, CSlit2, or Slit2ΔD2. (**g**) In vivo macropinocytosis was performed as described in Fig. 4g. Cells were labeled with FITC-conjugated anti-CD68 Ab (green) and TMR-labeled 70kDa dextran (red). Representative cells shown with XZ plane portrayed. Scale bar, 10 µm. Source data for (**b-d, f**) are provided as a Source Data file.

Supplementary Figure- 5

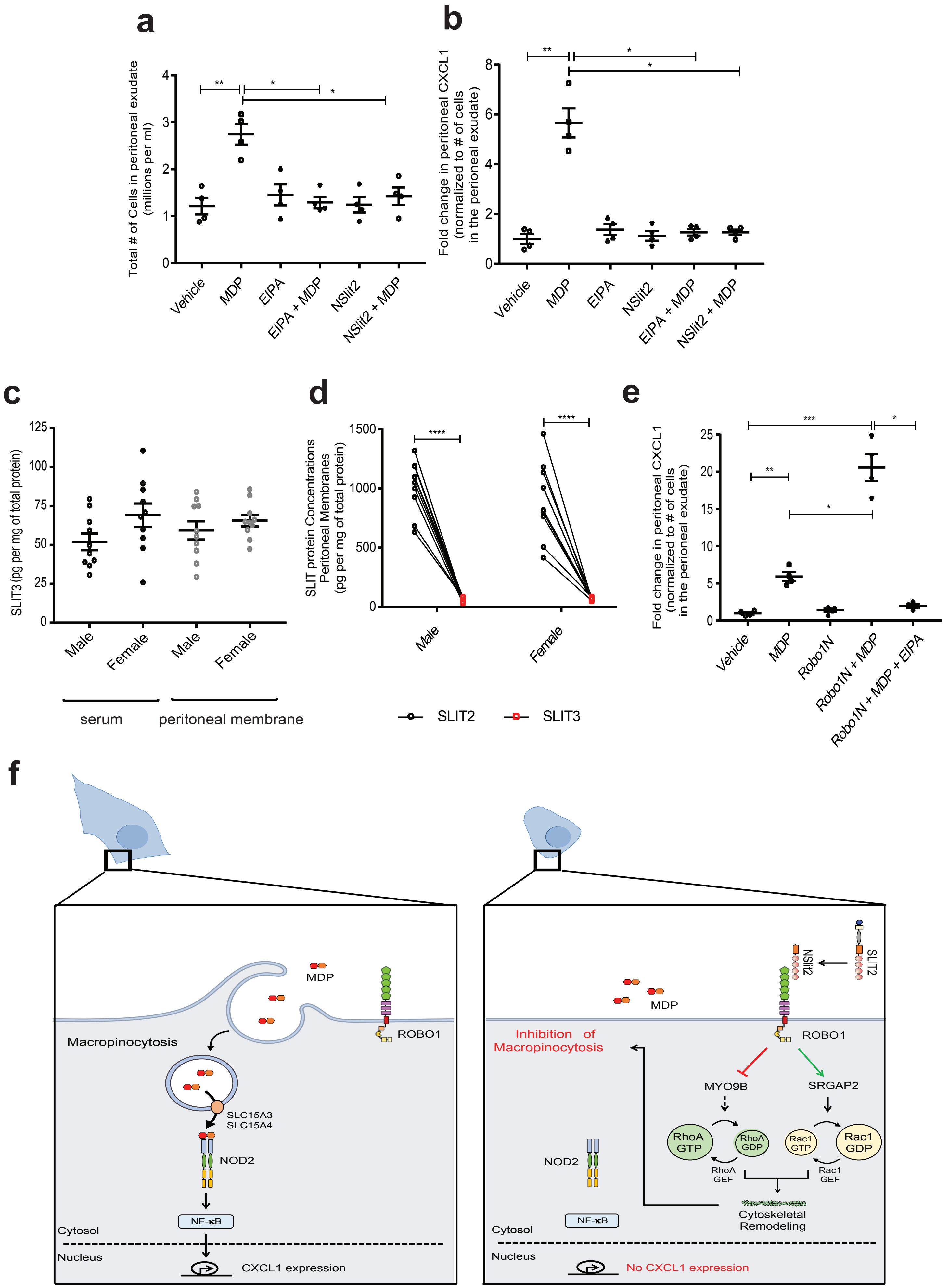

Supplementary Figure 5

(**a-e**) All data are presented as mean ± SEM. Comparisons between groups were made by Kruskal–Wallis ANOVA, followed by Dunn's multiple comparisons test. n= 4 animals per treatment group. (**a**) Animals were treated as described in Fig. 5e and cells in the peritoneal exudate were counted using a hemocytometer. ** $p = 0.0051$, vehicle vs MDP; * $p = 0.0404$, NSlit2 + MDP vs MDP alone; and * $p = 0.0124$, EIPA + MDP vs MDP alone . (**b**) Animals were treated as described in Fig. 5e. CXCL1 levels in the peritoneal exudate were measured using ELISA. ** $p = 0.0019$, vehicle vs MDP; * $p = 0.0455$, NSlit2 + MDP vs MDP alone; and * $p = 0.0316$, EIPA + MDP vs MDP alone. (**c**) Serum and peritoneal membrane samples were collected from mice as described in Fig. 5g. SLIT3 protein levels were measured using ELISA. (**d**) Comparison between peritoneal membrane levels of SLIT2 (from Fig. 5g) and SLIT3 (from Supplementary Fig. 5c). **** $p < 0.0001$, SLIT2 vs SLIT3. (**e**) Animals were treated as described in Fig. 5h. CXCL1 levels in the peritoneal exudates were measured using ELISA. *** $p = 0.0002$, Robo1N + MDP vs vehicle; ** $p = 0.0060$, vehicle vs MDP; and * $p = 0.0422$, Robo1N + MDP vs MDP alone. (**f**) Graphical summary of the effect of SLIT2-ROBO1 signaling on MDP-NOD2 signaling in macrophages. In the absence of SLIT2, MDP is taken up by macrophages via constitutive macropinocytosis. MDP enters the cytosol via SLC15A3/4 channels on the macropinosomal membrane and binds to the NOD2 receptors to activate NF-κB signaling. The nuclear translocation of activated NF-κB, in turn, stimulates the expression of pro-inflammatory cytokines, including CXCL1, *in vivo*. Endogenous SLIT2 is cleaved into a bioactive NSlit2 fragment, which binds to ROBO1 receptor expressed on the surface of macrophages. This recruits two GAPs, MYO9B and SRGAP2, to the cytosolic C-terminus of ROBO1. The ROBO1-MYO9B interaction inactivates RhoA GAP activity of MYO9B, whereas ROBO1-SRGAP2 interaction activates the Rac1 GAP activity of SRGAP2. As a result, there is activation of RhoA and inactivation of Rac1, ultimately leading to cytoskeletal remodeling in macrophages. NSlit2-

ROBO1-induced cytoskeletal remodeling prevents MDP uptake in macrophages by inhibiting constitutive and induced macropinocytosis, *in vitro* and *in vivo*. In turn, MDP-NOD2-induced CXCL1 upregulation is prevented. Source data for (**a-c, e**) are provided as a Source Data file.
