# Supplementary material for: SLIT2/ROBO1-signaling inhibits macropinocytosis by opposing cortical cytoskeletal remodeling
Source: Nat Commun. 2020 Aug 17;11:4112. doi: 10.1038/s41467-020-17651-1 (PMC7431850; doi:10.1038/s41467-020-17651-1)

Figure 1a

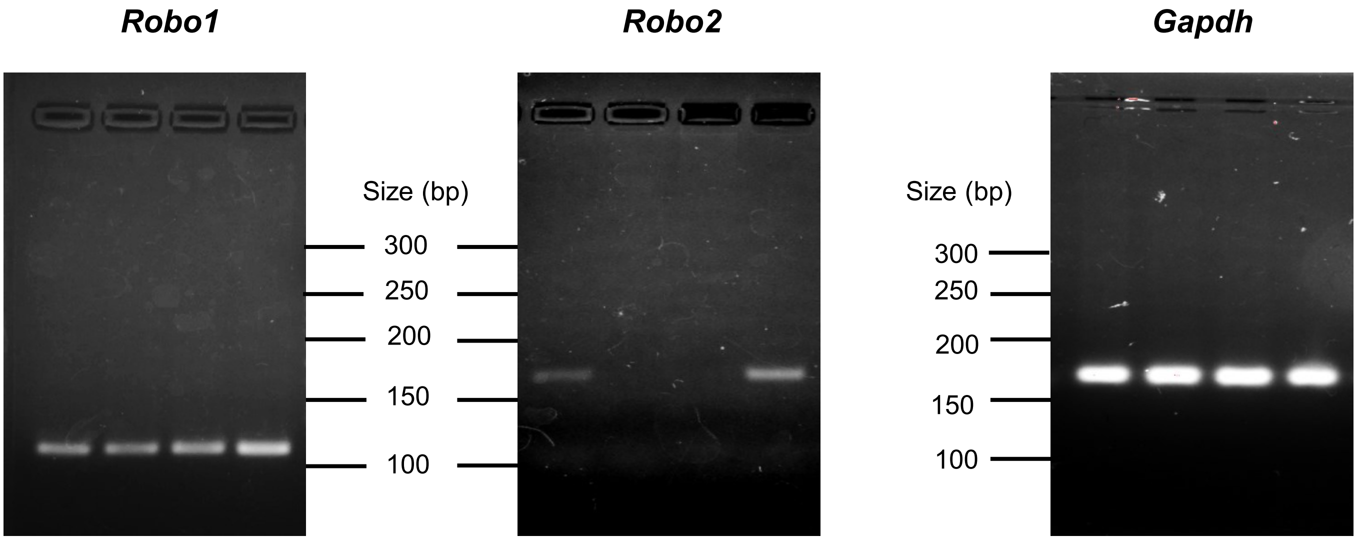

Supplementary Figure 1a

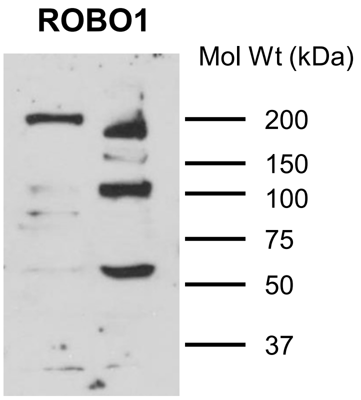

Fig. 1f and Supplementary Figure 1e

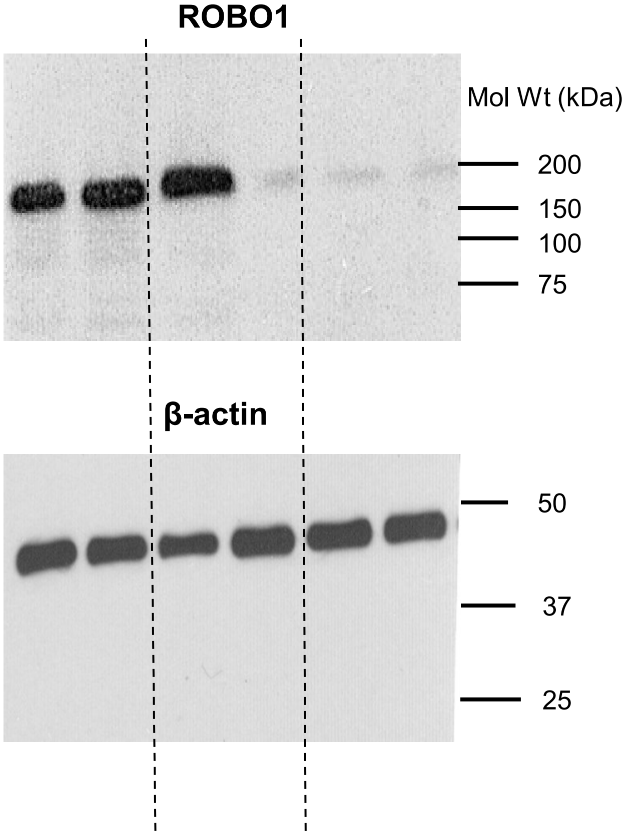

The part between the dotted lines is shown in Fig. 1f (upper panel) and the full gel is shown in Supplementary Fig. 1e

Supplementary Figure 2c

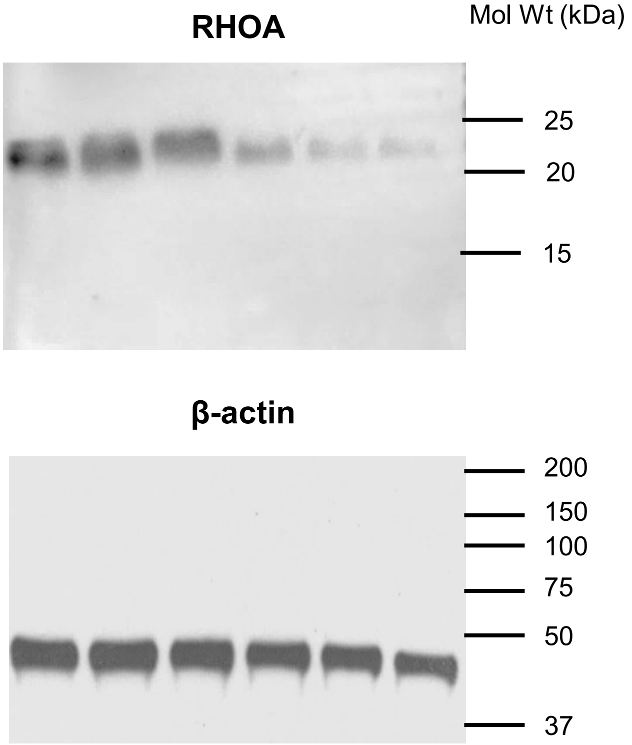

Supplementary Figure 3a

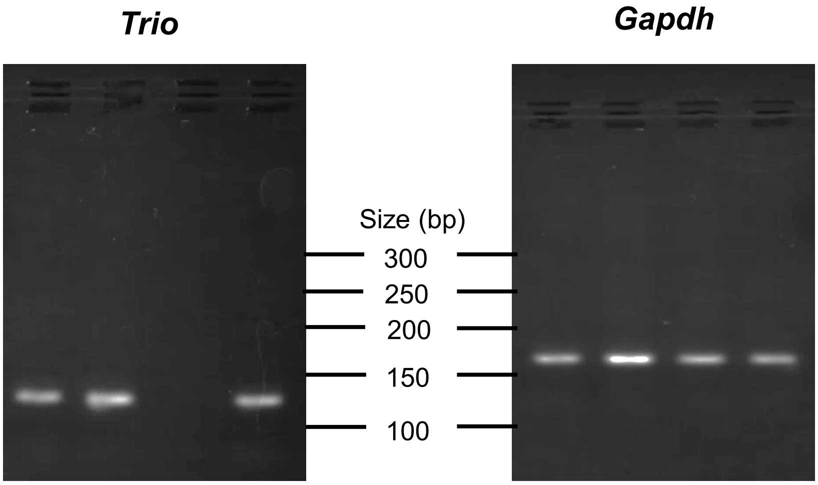

Supplementary Figure 3b

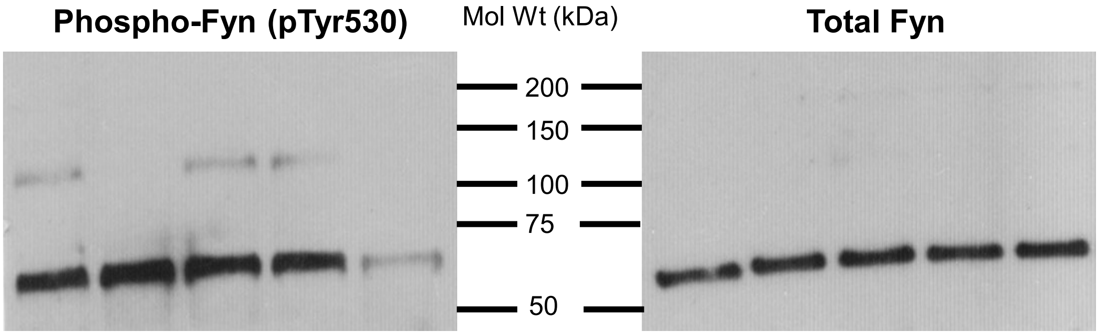

Supplementary Figure 3c

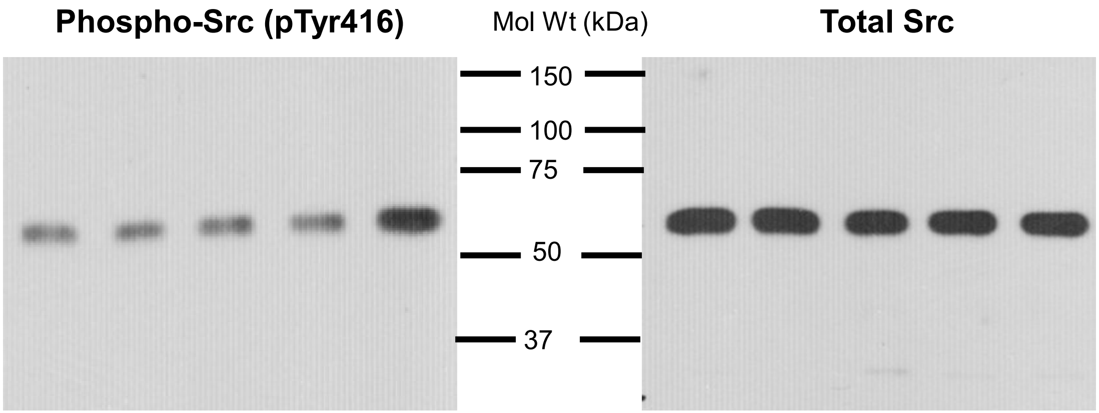

Supplementary Figure 3d

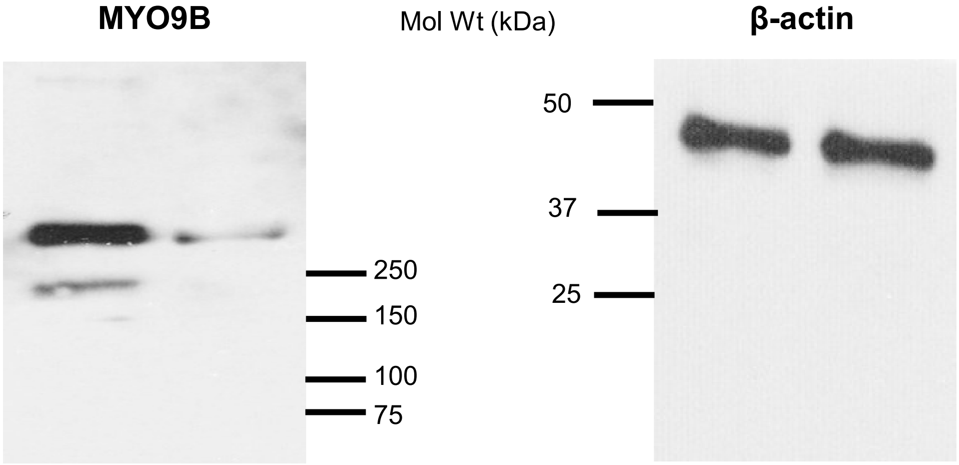

Supplementary Figure 4d

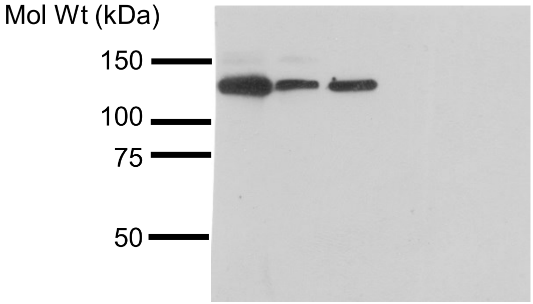

Figure 5c

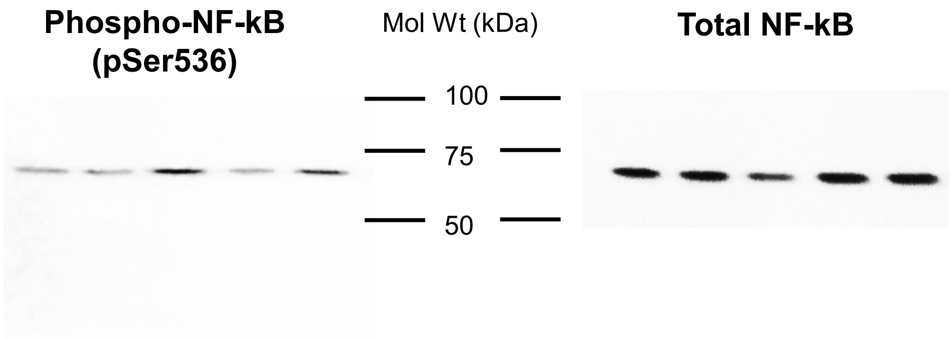

Supplement: Supplementary file 4 — Source Data [file 41467_2020_17651_MOESM4_ESM.zip › 41467_2020_17651_MOESM4_ESM/Source Data 3 (Blots and Gels)-1_ESM.pdf]
